# Correction: Gold plasmonic enhanced luminescence of silica encapsulated semiconductor hetero-nanoplatelets

**DOI:** 10.1039/d1na90082j

**Published:** 2021-09-22

**Authors:** Emilio Garcia, Christophe Arnold, Jean-Pierre Hermier, Michele D’Amico

**Affiliations:** Université Paris-Saclay, UVSQ, CNRS, GEMaC Versailles 78000 France jean-pierre.hermier@uvsq.fr; Nexdot 102 Avenue Gaston Roussel 93230 Romainville France michele.damico@nexdot.fr

## Abstract

Correction for ‘Gold plasmonic enhanced luminescence of silica encapsulated semiconductor hetero-nanoplatelets’ by Emilio Garcia *et al.*, *Nanoscale Adv.*, 2021, **3**, 4572–4578, DOI: 10.1039/D1NA00273B.

The authors regret that the following funding information was omitted from the Acknowledgements section of the original article.

Funding for this research was provided by: Association Nationale de la Recherche et de la Technologie (CIFRE No. 2018/1314).

The Royal Society of Chemistry apologises for these errors and any consequent inconvenience to authors and readers.

## Supplementary Material

